# Investigating the persistence of infant mortality and the Matthew effect in post-Soviet countries: evidence from nonlinear and Fourier-based time-series methods

**DOI:** 10.1017/S1463423626101534

**Published:** 2026-08-05

**Authors:** Nijat Gasim, Shahriyar Mukhtarov, Ayse Nur Sahinler, Aytakin Afandiyeva, Narmin Alakbarova

**Affiliations:** 1 Faculty of Medicine and Health Sciences, Qarabağ University, Azerbaijan; 2 International Scientific Research Center, Baku State University, Azerbaijan; 3 BEU-Scientific Research Center, Baku Engineering University, Azerbaijan; 4 University College, Korea University, South Korea; 5 Faculty of Business and International Relations, Vistula University, Poland; 6 Sustainable Development Center, Azerbaijan University of Architecture and Construction, Azerbaijan; 7 Department of Economics, Ankara Yildirim Beyazit University, Türkiye; 8 Department of Project Management, Azerbaijan University of Architecture and Construction, Azerbaijan; 9 Baku Higher Oil School, Azerbaijan; 10 Department of Economics, Azerbaijan State University of Economics (UNEC), Azerbaijan

**Keywords:** Fourier-based tests, Matthew effect, MCDA post-Soviet countries, non-linear, unit roots

## Abstract

This study investigates long-term trends in infant mortality rates (IMRs) in the case of 15 post-Soviet countries, with the aim of testing the Matthew Effect hypothesis, which suggests that inequality in health outcomes tend to persist or even widen over time. To assess these trends comprehensively, we employed various unit root tests, including traditional augmented Dickey–Fuller (ADF), non-linear (KSS, Kruse, Sollis, and Hepsag), and Fourier-based approaches (Fourier KPSS, Fourier ADF, flexible Fourier ADF, flexible fractional Fourier ADF, Fourier KSS, Fourier Kruse, and Fourier Sollis) to the data period from 1982 to 2022. Additionally, the multi-criteria decision analysis (MCDA) methods, such as equal weighting, entropy weighting, and TOPSIS, were utilized for a robustness check. The findings demonstrated that stationarity is validated in Armenia, Azerbaijan, Georgia, Kazakhstan, Kyrgyzstan, Lithuania, Latvia, Tajikistan, Turkmenistan, Ukraine, and Uzbekistan, thereby corroborating the Matthew Effect. Conversely, Belarus, Estonia, Moldova, and Russia exhibit non-stationary IMR series, indicating continued fluctuations or structural changes in their infant mortality patterns. The findings indicate that infant mortality has plateaued in several countries, pointing to stagnation in policy effectiveness. Conversely, some countries show dynamic change, possibly indicating responsive health systems. These insights provide important direction for country-specific health policy design to reduce IMR and health inequality more effectively.

## Introduction

The infant mortality rate (IMR) is the ratio of the number of newborns who pass away prior to reaching their first birthday to the total number of live births (Tomlin *et al*., 2021). The IMR is an essential indicator of a population’s health and welfare because it reflects the standard of available medical treatment, highlights existing inequalities, predicts potential future health outcomes, influences economic growth, and serves as a primary concern for global health.

Significant advancements have been achieved globally in decreasing mortality rates. Since 2000, the worldwide under-five mortality rate has decreased by 52%, demonstrating a significant collaborative effort by governments, donors, and communities (UN IGME, 2025). This advancement signifies the preservation of millions of lives – children who have been given the opportunity to develop, acquire knowledge, and contribute to their communities and society at large. Children are the cornerstone of future economies; investing in their survival will provide benefits for communities both now and in subsequent generations. Nonetheless, the latest data about under-five mortality unequivocally indicate that the endeavour to eradicate all avoidable child fatalities remains incomplete (UN IGME, 2025). In 2023, 4.8 million children, with a range of 4.5–5.3 million, perished before their fifth birthday, mostly due to avoidable causes. This encompasses 2.3 million (ranging from 2.1 to 2.6 million) fatalities during the newborn era (i.e., ages 0–27 days) and 2.5 million (ranging from 2.3 to 2.9 million) fatalities among children aged 1–59 months. Alongside the burden of under-five mortality, there were 2.1 (2.0–2.4) million fatalities among individuals aged 5–24 years, which included 0.9 (0.9–1.0) million deaths among teenagers aged 10–19 years (UN IGME, 2025).

**Figure 1.**
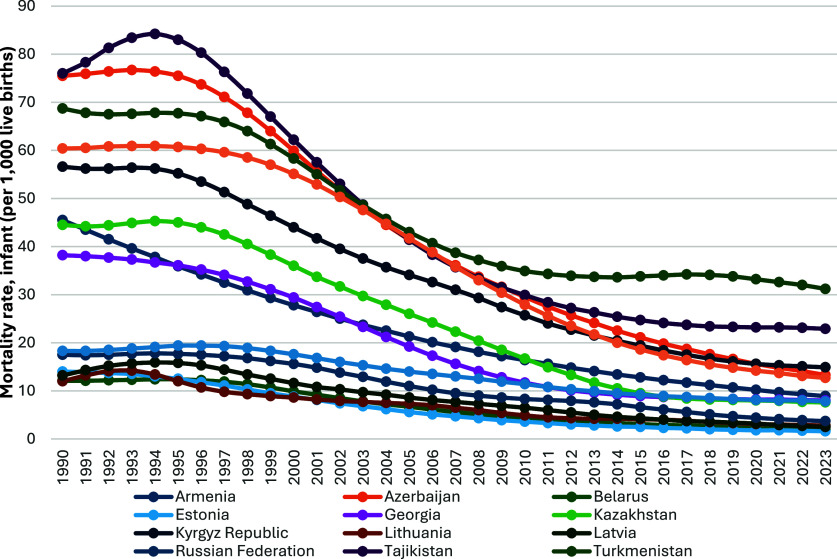
Figure 1 long description. Infant mortality rate (per 1,000 live births) in post-Soviet countries.

The Sustainable Development Goals state that all nations lower the under-five mortality rate (U5MR) to a maximum of 25 deaths per 1,000 live births by 2030 and decrease the neonatal mortality rate to no more than 12 deaths per 1,000 live births by 2030 (UN IGME, 2025). For achieving the SDG targets, effective national policy, global collaboration, and concentrated domestic investments in child health are essential. In this perspective, it is important to examine trends in child mortality in post-Soviet countries. Figure 1 illustrates the trajectory of child mortality in 15 post-Soviet nations. Figure 1 illustrates a significant decline in infant mortality across all post-Soviet nations in recent years. Additinally, the IMR in the post-Soviet countries exhibits considerable variation across different nations. In 2023, some countries, such as Estonia and Belarus exhibited relatively low rates of 1.6 and 1.9 deaths per 1,000 live births respectively, while others showed significantly higher rates, such as 22.9 and 31.2 deaths per 1,000 live births for Tajikistan and Turkmenistan, respectively (World Bank, 2025).

Long-term research of the dynamics of IMRs across countries is needed due to the importance of this variable as an indication of a country’s welfare, the variable’s dynamic response to health or education policies and other exogenous shocks, and the significant cross-country disparities in the IMR (Gil-Alana *et al*., 2017). Different studies reported that the IMR is influenced by access to healthcare (Schoeps *et al*., 2011; Gruber *et al*., 2014), social-economic status (SES) (MacMahon *et al*., 1972; Hobcraft *et al*., 1984; Van Malderen *et al*., 2019), maternal education (Green and Hamilton, 2019; Kiross *et al*., 2019), environmental factors (Mettetal, 2019; Rasoulinezhad *et al*., 2020; Shobande, 2020), and nutrition (Uddin *et al*., 2009; Lassi *et al*., 2020). In addition, individuals with higher SES tend to be healthier than those with lower SES. This is known as the socio-economic gradient in health. Studies that include measurements of income level reveal that it is significantly connected to health outcomes (Ettner, 1996; Frijters *et al*., 2005; Vanzella-Yang and Veenstra, 2021). In general, countries with higher average incomes also tend to have lower IMRs. This decrease is because wealthier countries can invest more in healthcare, nutrition, and other factors that contribute to infant health and well-being (Semyonov *et al*., 2013).

Based on the law of diminishing returns, one would expect convergence of the health status because the benefit gained will start to decline, eventually reaching a point where further input will not result in any significant increase in output or benefit (Gächter and Theurl, 2011). Even if individuals or nations continue to make significant efforts to improve their health, they may eventually encounter a point where their gains diminish. At a certain stage, additional health expenditures may generate diminishing returns compared to earlier investments. To reduce infant mortality, countries often begin with highly cost-effective interventions; however, once substantial progress is made, additional improvements may require investment in less cost-efficient measures (Bishai and Poon, 2001; Bishai *et al*., 2007). On the other hand, several researchers argue that pepople with poor baseline health experience limited improvement over time, while those with better initial health tend to experience substantial gains (Joseph, 1989; Dzakpasu *et al*., 2000). This is called the Matthew effect in the IMR. The idea of the Matthew effect was first proposed by sociologist Merton (1968). Merton (1968) named the effect after a passage from the Bible (Matthew 25:29), which states, ‘*For whoever has will be given more, and they will have an abundance. Whoever does not have, even what they have will be taken from them’.* Thus, it is based on the fact that healthcare services tend to be concentrated in higher-income countries where demand is greater. In contrast, countries with lower income and more health requirements often have fewer healthcare resources accessible. Applied to infant mortality, this effect implies that countries with initially low IMR and better health infrastructure are more likely to sustain improvements, whereas countries with high IMR face greater challenges in reducing mortality rates. This mechanism underpins the theoretical foundation of the present study.

Infant mortality is widely recognized as a vital indicator for assessing both the performance of national health systems and the broader course of socio-economic development (UN IGME, 2025). This approach is particularly important in the case of post-Soviet countries. After the collapse of the Soviet Union, the development of health systems in the region has shown differences, and while institutional reforms and improved service quality have been implemented in some countries, in others, social protection systems have weakened and inequalities in access to health have persisted (Semenova *et al*., 2024). Therefore, the stagnation or weak dynamics observed in the IMR indicators in the region should be explained not only by macroeconomic indicators, but also by the inclusiveness of development and the effectiveness of social policies. Thus, the investigation of child mortality in post-Soviet nations has significant scientific and practical relevance for discovering socio-economic disparities, evaluating health system efficiency, comprehending the consequences of the transitional phase, and facilitating comparisons across countries. The focus on post-Soviet countries stems from their shared historical experience of systemic political and economic transition following the dissolution of the Soviet Union, despite which they have followed markedly divergent paths in both health sector reforms and socio-economic progress. By exploring these divergences, the study seeks to uncover how cross-country differences have shaped infant mortality patterns and to offer a comparative perspective on their long-term trajectories.

Considering above-mentioned facts, this study aims to explore following critical questions: (i) Is the Matthew Effect observed in IMRs in post-Soviet countries? (ii) What are the long-term trends in IMRs in post-Soviet countries? Therefore, this artticle investigates the long-term dynamics of infant mortality while simultaneously testing the validity of the Matthew Effect across all post-Soviet countries, utilizing a range of unit root tests – including the traditional augmented Dickey–Fuller (ADF), as well as non-linear and Fourier-based approaches.

The contribution of this paper are as follows: (i) To the best of our knowledge, this is the first study to examine the long-term dynamics of infant mortality, as well as testing the validity of the Matthew Effect across all post-Soviet countries. (ii) Methodologically, this is one of the few studies to employ a wide range of unit root tests including the traditional ADF, non-linear and Fourier-based approaches. This approach offers an important advantage in identifying structural breaks and cyclical fluctuations that cannot be detected by conventional linear tests. (iii) A key feature that distinguishes this study from previous studies is its use of multi-criteria decision analysis (MCDA) in the decision-making process, relying on the outcomes of 12 different unit root tests rather than a simple numerical majority (e.g., 5/7 or 3/9). This approach enables a more holistic and balanced analysis by incorporating the varying sensitivities and methodological perspectives of the tests. Overall, the findings of this study can be useful for formulating long-term health policies and hold significant implications for policymakers addressing infant mortality in post-Soviet countries.

## Literature review

The IMR is arguably the most frequently used development indicator in the literature, which is often used as a measure of the overall health status of a country, as well as its socio-economic development (Gonzalez and Gilleskie, 2017). The IMR measures a consequence of the development process rather than an input, such as health spending. It is also recognized to stem from other variables, including the accessibility of maternal and child health services (such as prenatal care), immunization rates, family income, and food security, as well as the provision of clean drinking water and basic sanitation (UK Data Service, 2008). Thus, IMR can be used as an important tool in assessing the extent to which economic growth can contribute to the development of human well-being (UN IGME, 2025).

In addition to this, the increasing body of empirical evidence suggests that IMR is closely linked with income levels and inequality and the distributive aspects of development. For instance, it has been found that the inequality-adjusted human development index (IHDI) was more effective in explaining the variations in IMR levels in comparison with the standard HDI. This underlines the significance of distributive aspects in the context of development and health outcomes (Ruiz *et al*., 2015). Similarly, maternal education has been identified as a key determinant of infant mortality reduction. This underlines the significance of social and human capital dimensions of development (Schellekens, 2021). Thus, it can be seen that these findings collectively indicate that economic growth alone is insufficient to reduce IMR unless accompanied by improvements in social policy, education, and equitable access to healthcare (Hanmer *et al*., 2003).

Nonetheless, the IMR is not a perfect indicator. The research indicates that variations in the definition of live birth, the quality of death registration, and statistical reporting might result in measurement inaccuracies in cross-national comparisons (Gonzalez and Gilleskie, 2017). Consequently, the IMR is often seen as a ‘imperfect yet informative’ development metric. In this contex, infant mortality has been extensively explored in literature as a measure of public health advancement and a critical indication of health disparities. A key discourse on the topic is the Matthew Effect posited by Joseph (1989). The Matthew Effect was first brought into sociological literature by Merton (1968) to explain the phenomena of cumulative advantages and the exacerbation of disparities in scientific output. This notion, applied to health, posits that societies with superior beginning health indicators see more rapid improvement over time, while disadvantaged groups advance at a comparatively slower pace or fall behind (Joseph, 1989). This approach, when considered in terms of infant mortality, can be interpreted as countries or social groups with low initial IMR recovering faster, thus reinforcing their advantages, while those with high IMR stagnate or progress slowly, thus perpetuating their disadvantages (Dzakpasu *et al.*, 2000). Therefore, the Matthew effect hypothesis is based on the prediction that, in infant mortality, countries with low initial mortality progress faster, while countries with high mortality lag behind. This notion theoretically suggests that health inequalities will not spontaneously close but may become persistent without active intervention. Consequently, infant mortality reflects not only biomedical factors but also social inequalities and cumulative advantage/disadvantage processes. The presence of stationarity in the IMR series would indicate that countries with initially low infant mortality have maintained their advantage over time, while those with high rates remain persistently disadvantaged – thus supporting the Matthew Effect. Conversely, if the IMR series is found to be non-stationary, this would imply that health policy interventions have altered the trajectory of the series, enabling countries to reduce disparities and gradually shift away from persistence, thereby refuting the Matthew Effect. In this context, time series methods have been used to test the stationarity of IMR series in some studies. Bishai (1995) applied the ADF test to IMR data in the case of Sweden for the data period of 1800–1989, in the case of the UK for the data period of 1839–1989, and in the case of the US for the data period of 1915–1989. The findings indicated that the null hypothesis of a unit root could not be rejected in any of these economies, implying non-stationarity. Similarly, Dreger and Reimers (2005) examined IMR stationarity in the case of 21 OECD countries from 1975 to 2001 but did not reach definitive conclusions regarding the stationarity of the series. Silva (2007) conducted panel stationarity tests that accounted for structural breaks in IMR in tha case of Australia, using data period from 1911 to 2002. His analysis found that even after considering structural breaks, the unit root hypothesis could not be rejected across all Australian states. Moreover, later research has used time series methodologies to empirically examine the Matthew Effect. Bishai *et al.* (2007) tested the Matthew Effect hypothesis using the DFGLS unit root test on IMR data from 1870 to 1988. The study revealed that the IMR series in most countries was non-stationary, and the Matthew Effect did not hold. Aguirre and Vela-Peón (2015) examined the regional disparities in the decline of infant mortality to evaluate the Matthew Effect hypothesis in Mexico. The Spearman and Kendall rank correlation coefficients were employed to evaluate the hypothesis. The estimation results do not confirm the Matthew Effect.

In addition, several researchers, such as Diebold and Inoue (2001) and Granger and Hyung (2004), argue that fractional integration may be a spurious outcome caused by unaccounted structural breaks. Later, Bishai and Opuni (2009) examined IMR trends in 18 countries and found that only one country experienced an exponential decline in IMR, whereas for the other 17, the slope of the IMR variable did not conform to a logarithmic transformation.

Conversely, Erdogan *et al*. (2013) examined IMR stationarity in 25 high-income OECD countries from 1970 to 2007, concluding that these series were stationary. Caporale and Gil-Alana (2014) applied the fractional unit root test to IMR data from 24 countries, allowing for fractional levels of integration in time series analysis. Their findings indicated that integration levels varied across countries. Another study by Gil-Alana *et al.* (2017) examined IMR time series properties for 37 countries, concluding that degrees of linearity and integration differed among the series. They argued that IMR convergence across countries was unlikely due to these integration disparities. Aditionally, Genowska *et al*. (2021) discovered that, mortality rates for infants under one year of age dramatically declined across all nations, with a sustained convergence found between the Baltic states and the EU-28. Esmaeilzadeh *et al*. (2021) conducted a trend analysis of IMRs across various regions of the WHO from 1990 to 2017. Our findings indicated a declining trend in IMR across all WHO regions. In a more recent study, these discussions are conducted with more nuance. For example, Yılancı and Canpolat Gökçe (2021) analysed the IMR characteristics of 33 countries using the flexible Fourier augmented Dickey–Fuller (FFADF) test, which incorporates multiple smooth structural breaks. The results demonstrated that Fourier terms were significant in 22 countries, indicating the need to consider multiple smooth changes when testing for stationarity. Consequently, the FFADF unit root test was applied to those 22 countries, while the standard ADF test was used for the remaining cases. The findings showed that IMR series in 20 countries were stationary. Javanshirova (2024) analysed the stationarity levels of the IMR utilizing non-linear unit root tests (KSS, Sollis, and Kruse) to data period from 1982 to 2022 in the case of Azerbaijan. The results revealed that IRM is stationary. In addition, Mehdi (2019) for Pakistan, and Odjesa and Ighodaro (2021) for Nigeria found that IRM is stationary, using the ADF unit root test while Bahuguna *et al*. (2025) revealed that IMR are non-stationary in the case of India, utilizing the ADF and KPSS unit root tests. Also, Ogundunmade *et al*. (2023) revealed that IMR are non-stationary in the case of Nigeria, using ADF unit root test.

As can be seen from the literature review, child mortality has been studied from various aspects in different countries. However, no study has been conducted to examine the long-term trends of child mortality rates and to test the validity of the Matthew Effect in the case of all post-Soviet countries. The main aim of our study is to fill this gap in the literature by using various unit root tests, including traditional ADF, non-linear and Fourier-based approaches, to examine the long-term trends of child mortality rates and to test the validity of the Matthew Effect in 15 post-Soviet countries. In addition, our study differs from prior research by including multiple unit root tests with MCDA methodologies, including Equal Weight, Entropy Weight, and TOPSIS. This facilitates a more comprehensive and unbiased analysis that considers the varying sensitivities and methodological frameworks of the tests. Therefore, the study’s results are valuable for developing long-term health strategies and have considerable significance for policymakers tackling infant mortality in post-Soviet nations.

## Methodology and data

### Methodology

This study examines whether IMRs in post-Soviet countries are stationary, consistent with the Matthew Effect hypothesis. The fundamental data-generating method of the IMR series may fluctuate over time and within nations, possibly altering under various structural regimes. Exclusively relying on traditional unit root tests to assess the stationarity of such a series may result in erroneous findings. In acknowledgment of this constraint, a variety of unit root tests – each addressing distinct characteristics of time series behaviour – has been used in this work to guarantee a more thorough and resilient evaluation. This multi-test technique aims to improve the dependability of the results and function as a robustness check for the econometric study (Gasim *et al*., 2025).

The presence of structural breaks in time series is an important factor that directly influences analyses of whether the series is stationary. In the literature, it is frequently discussed that traditional Dickey–Fuller (1979) type unit root tests do not account for these structural breaks and therefore suffer from low test power.

(1) $$\Delta Y_{t}=\beta_{1}+\beta_{2}t+\rho Y_{t-1}+\sum_{k=1}^{k}\theta_{k}\Delta Y_{t-k}+u_{t}$$

(2) $$\Delta Y_{t}=\beta_{1}+\beta_{2}t+\rho Y_{t-1}^{3}+\sum_{k=1}^{k}\theta_{k}\Delta Y_{t-k}+u_{t}$$

(3) $$\Delta Y_{t}=\beta_{1}+\beta_{2}t+\rho Y_{t-1}+\phi_{1}\sin\left(\frac{2\pi kt}{T}\right)+\phi_{2}\cos\left(\frac{2\pi kt}{T}\right)+\sum_{k=1}^{k}\theta_{k}\Delta Y_{t-k}+u_{t}$$

Equations (1)–(3) present the baseline specifications of the ADF (Dickey and Fuller, 1981), KSS, and Fourier ADF unit root tests. The other tests applied in this study follow a similar general structure but differ in their functional forms and underlying assumptions.

A well-known limitation of conventional unit root tests is their low power in the presence of structural breaks, which may lead them to incorrectly fail to reject the null hypothesis of a unit root even when the series is stationary (Phillips and Xiao, 1998). This limitation increases the risk of misinterpreting stationary processes as non-stationary, particularly in economic time series.

To address this problem, Perron (1989) developed one of the earliest unit root tests with structural breaks by incorporating dummy variables to identify break points. However, this approach assumes that structural changes occur instantaneously, which is rarely the case in economic processes that typically evolve gradually. To allow for smoother transitions, Leybourne *et al*. (1998) introduced a logistic smooth transition function into the unit root framework, and Harvey and Mills (2002) extended this approach by considering two smooth breaks.

Subsequent research emphasized non-linear dynamics. Kapetanios *et al*. (2003) proposed the KSS test, which assumes that the time series follows a first-order exponential smooth transition autoregressive (ESTAR) process. Building on this, Sollis (2009) incorporated both logistic and exponential transition functions and allowed for asymmetric mean reversion depending on the sign of shocks. Kruse (2011) further extended the KSS framework by relaxing the restriction on the localization parameter, while Hepsag (2021) combined smooth breaks with non-linear ESTAR dynamics, providing an even more flexible treatment of structural changes.

In this study, Fourier-based approaches were also employed in conjunction with traditional unit root tests. These tests have several important advantages over traditional unit root and structural break tests. The traditional unit root tests like ADF and Phillips-Perron are based on linear assumptions and do not take into account any structural breaks. Therefore, these tests may have low power and may not reject the null hypothesis of a unit root even in the presence of breaks (Perron, 1989). This problem is partially addressed by structural break tests like Perron (1989), Zivot and Andrews (1992), and Lee and Strazicich (2003, 2004). However, these tests necessitate prior specification of the quantity, timing, and functional form of breaks, which may result in pre-test bias and model misspecification (Maddala and Kim, 2010; Enders and Lee, 2012). The Fourier-based unit root tests overcome these problems by allowing for the inclusion of low-frequency sine and cosine components that are able to capture smooth and unknown forms of structural breaks without any prior knowledge of the number and timing of breaks. Therefore, these methods are better able to capture smooth and multiple forms of structural breaks. This increases test power and robustness. An outstanding contribution to the development of unit root and break tests is made by Becker *et al*. (2006), who proposed the Fourier KPSS test. The Fourier ADF, Flexible Fourier ADF (Enders and Lee, 2012), Flexible Fractional Fourier ADF (Omay, 2015), Fourier Sollis (Ranjbar *et al*., 2018), and Fourier Kruse (Güris, 2019) are also important developments in this tests.

The number of Fourier frequencies is denoted by the parameter *k*. We limited *k* to the range of 1–5 in accordance with Becker *et al.* (2006), which has become standard in the literature because it offers enough flexibility to capture smooth structural shifts without over-parameterization or power loss in finite samples. The sum of squared residuals was minimized in order to determine the optimum frequency.

For the majority of tests employed – such as ADF, KSS, Kruse, and Sollis – critical values corresponding to the constant-and-trend specification are readily available in their original studies. However, the Fourier ADF test represents an exception in this regard, as the standard literature provides critical values only for the constant-only version. To address this gap, we adopted the simulated critical values proposed by Hepsağ (2022), which were specifically developed for the Fourier ADF test with both constant and trend.

Lag length selection was conducted in line with Schwert’s (1989) widely applied rule, Kmax = 12 × (T/100) ^ 1/4.

where T denotes the sample size. After setting this maximum lag, the optimal lag order was selected using the Akaike information criterion (AIC) and the Schwarz Bayesian Criterion/Bayesian information criterion (SBC/BIC). In cases where AIC and BIC suggested different lag orders, priority was given to the more parsimonious BIC, consistent with standard econometric practice. This approach ensures that residuals are free of serial correlation while preserving degrees of freedom.

The application of this wide set of unit root tests to the infant mortality series of post-Soviet countries allows for a robust and comprehensive analysis, ensuring that persistence results are validated across different methodological frameworks.

In the context of unit root testing, if the absolute value of the test statistic exceeds the corresponding critical value, the null hypothesis of non-stationarity is rejected. This outcome implies that the time series is stationary, which, in turn, provides empirical support for the presence of the Matthew Effect. While this logic applies to the eleven unit root tests employed in the analysis, it is important to note that the Fourier KPSS (FKPSS) test follows a reversed hypothesis structure. In the FKPSS framework, the null hypothesis assumes stationarity, and thus failure to reject it indicates that the series is stationary and the Matthew Effect remains valid. Consequently, the interpretation of FKPSS results requires special attention to its distinct null formulation to avoid misclassification. In the context of Fourier-based unit root tests, if the series is found to be non-stationary, the significance of the F-statistic – which tests the relevance of the Fourier terms – becomes inconsequential. However, if the series is determined to be stationary, then the Fourier terms must be statistically significant; otherwise, the classical version of the test (without Fourier terms) should be considered valid instead. For example, if the FSollis test indicates stationarity but the associated F-statistic is not significant, then the results of the standard Sollis test would be more appropriate for interpretation. Beyond the differences in hypothesis structures, each of the tests also evaluates stationarity under different assumptions and examines the structural properties of the series from various aspects. Fourier-based tests are better able to capture periodic variations in the series, while non-linear tests are more sensitive to situations without abrupt changes.

#### Robustness check

An integrated and robust framework for evaluating the presence of the Matthew Effect is constructed using three multi-criteria decision-making analysis (MCDA) methods – Equal Weighting, Entropy Weighting, and TOPSIS (Technique for Order Preference by Similarity to Ideal Solution). To our knowledge, this represents the first attempt to synthesize the outcomes of twelve distinct unit root tests through an MCDA-based approach in the context of post-Soviet countries. This combination enables a more transparent and multidimensional interpretation of the stationarity evidence. Each of these methods offers a unique lens through which the results of the twelve unit root tests can be interpreted more systematically and transparently.

Equal Weighting is the most straightforward of the three. It assumes that all unit root tests provide equally valuable information and therefore assigns the same importance to each of them. For every country, the number of stationary outcomes is averaged across all tests. This method is particularly useful for providing an initial overview and ensuring that no test is arbitrarily prioritized over another (Booysen, 2002). While it is simple, it also helps detect general patterns and flag countries that consistently appear to exhibit the Matthew Effect across multiple tests.

On the other hand, Entropy Weighting moves beyond this simplicity and introduces a more data-sensitive approach. It evaluates the informational contribution of each test by measuring the variability – or entropy – of the test results across countries. Tests that generate more heterogeneous outcomes are considered more informative and receive higher weights. This way, redundancy is penalized, and tests that better distinguish between countries have a greater influence on the final score. Entropy weighting is particularly valuable when the goal is to reduce bias arising from overly uniform tests and to emphasize those that capture unique signals in the data (Hainmueller, 2012).

In addition, TOPSIS offers a more sophisticated ranking mechanism. Unlike the previous two, TOPSIS does not simply aggregate or weigh test results. Instead, it positions each country within a multidimensional space defined by all test outcomes and measures its relative closeness to an ‘ideal’ point (where all tests indicate stationarity) and an ‘anti-ideal’ point (where all tests indicate non-stationarity) (Hwang and Yoon, 1981). The closer a country is to the ideal point, the more consistently its IMR series is found to be stationary across tests, thereby suggesting stronger evidence in favour of the Matthew Effect. This method is particularly useful when trying to rank countries based on nuanced differences in test outcomes and provides an intuitive sense of how far each case lies from extreme scenarios.

The selection of these three methods is deliberate: Equal Weighting provides a neutral benchmark, Entropy Weighting introduces an information-sensitive adjustment, and TOPSIS delivers a ranking-based perspective. By triangulating these complementary approaches, the study ensures robustness and avoids dependence on any single decision-making logic. This combination therefore allows us to balance simplicity, statistical informativeness, and comparative ranking, offering a more reliable synthesis of the diverse unit root test results. Taken together, these three methods allow for a rich, multi-layered interpretation of the unit root test results. They help mitigate the limitations of individual tests, ensure robustness in classification, and provide a well-rounded basis for evaluating the presence of the Matthew Effect across post-Soviet countries. By combining both equal and data-driven perspectives, the analysis becomes not only more methodologically rigorous but also more insightful and transparent.

### Data

In order to implement the empirical methods outlined above, the dataset used in the analysis must first be introduced. Accordingly, the IMR variable serves as the core focus of the study, and its definition, data sources, and temporal coverage are provided in Table 1.

**Table 1.** Variable definition Table 1 long description.

Variable	Definition	Period	Source
IMR	Infant mortality rate, (per 1,000 live births)	1982–2022	World Bank, World Development Indicators (World Bank, 2025)

*Note*: IMR denotes infant mortality rate, expressed as the number of infant deaths per 1,000 live births. Data are sourced from the World Bank World Development Indicators and cover the period 1982–2022.

Analysing the fundamental statistical characteristics of the dataset provides important information about the distribution and variability of the variables. In this context, the descriptive statistics for IMRs are summarized in Table 2. The table includes the mean, median, minimum, maximum, standard deviation, Jarque–Bera (JB) statistic, and probability (Prob) values. These statistics make it possible to evaluate the measures of central tendency and distributional properties of the variables, thereby revealing the basic structure of the series before econometric analyses.

**Table 2.** Descriptive statistics Table 2 long description.

Countries	Mean	Median	Min	Max	Std. Dev.	Jarque–Bera (prob)
ARM	27.9	24.6	9.19	63.4	15.1	3.43(0.18)
AZE	52.2	53.6	16.1	86.5	24.3	4.31(0.12)
BLR	8.32	8.50	1.90	15.7	4.54	4.01(0.13)
EST	8.16	7.30	1.50	16.5	5.23	4.37(0.11)
GEO	26.2	27.5	8.30	46.4	14.0	4.81(0.09)
KAZ	30.6	32.5	8.69	54.8	15.6	3.97(0.14)
KYR	39.5	38.4	15.4	74.0	17.5	2.48(0.20)
LAT	9.66	10.3	2.80	15.9	4.39	3.64(0.16)
LIT	8.35	7.90	2.90	15.1	4.08	3.39(0.18)
MDA	22.3	21.8	12.2	37.1	8.26	4.37(0.11)
RF	12.8	13.9	3.80	21.3	5.59	3.74(0.15)
TJK	60.1	58.4	26.7	98.6	25.1	4.63(0.10)
TKM	52.9	51.6	35.0	85.5	15.5	3.21(0.20)
UKR	13.5	14.3	7.00	21.1	4.39	3.40(0.18)
UZB	42.5	47.1	11.9	80.4	20.2	2.76(0.25)

*Note*: Mean = average infant mortality rate (IMR, deaths per 1,000 live births); Std. Dev. = standard deviation. Jarque–Bera (JB) test values are reported with probability levels in parentheses. A higher JB value with a probability less than 0.05 indicates rejection of the null hypothesis of normality.

Table 2 reports the descriptive statistics of IMR for the 15 post-Soviet countries over the period 1982–2022. The statistics include mean, median, minimum, maximum, standard deviation, and the JB test for normality. The data were analysed in their original form without logarithmic transformation, as infant mortality is already expressed in relative terms (deaths per 1,000 live births). The sample period of 1982–2022 was selected because consistent data for all fifteen countries are available only for this timeframe, ensuring comparability across cases.

The results highlight considerable cross-country differences. Tajikistan (60.1), Azerbaijan (52.2), and Turkmenistan (52.9) exhibit the highest average IMRs, while Estonia (8.16), Belarus (8.32), and Lithuania (8.35) record the lowest. Standard deviations are also large in Tajikistan (25.1), Azerbaijan (24.3), and Uzbekistan (20.2), indicating substantial fluctuations in mortality dynamics, whereas Belarus (4.54), Estonia (5.23), and Ukraine (4.39) demonstrate more stable trends.

The comparison between mean and median shows that in several cases (e.g., Azerbaijan, Uzbekistan, Turkmenistan), the mean exceeds the median, suggesting right-skewed distributions where historically high mortality values continue to influence the average. This points to a gradual decline in infant mortality over time.

The JB test statistics indicate that for most countries, the null hypothesis of normality cannot be rejected at the 5% level, as probability values generally exceed 0.05. However, relatively high JB statistics for Georgia (*p* = 0.09) and Tajikistan (*p* = 0.10) suggest potential deviations from normality. These findings support the combined use of parametric (e.g., ADF, KSS) and non-parametric (e.g., Sollis, Kruse, Hepsag, Fourier-based) unit root tests in the subsequent analysis.

In sum, the descriptive statistics reveal not only differences in the level of infant mortality across post-Soviet countries but also variations in volatility and distributional properties. These results underline the importance of employing a diverse set of econometric tests to assess the persistence of infant mortality dynamics.

## Empirical results and discussions

This section provides an analysis and discusses the results derived from the tests outlined in the methodology section. First, a trend analysis was conducted, and the results are presented in Appendix 1, Table A1. According to the trend analysis findings, the IMR series for all countries were found to contain a trend. Before applying the unit root tests, linearity tests proposed by Harvey and Leybourne (2007) and Harvey *et al*. (2008) were conducted to determine whether the series exhibit linear or non-linear behaviour. The results of linearity tests are presented in Appendix, Tables A2. Consequently, the unit root tests were applied within a model framework that includes both a constant and a trend. Next, to determine the stationarity characteristics of the series, unit root tests were carried out. In this context, the ADF and Fourier ADF (FADF) tests were performed first. Table 3 summarizes the results of the traditional ADF test and the alternative FADF test.

**Table 3.** Results of ADF and fourier ADF Table 3 long description.

	ADF	FADF (2010)		
	C&T	C&T		
Countries	Test stat	*k*	Test stat	*F*
ARM	−4.3112***	1	−4.6943**	23.035***
AZE	−5.3783***	1	−7.0944***	645.25***
BLR	−2.9723	1	−1.3679	305.83
EST	−3.9890**	1	−1.7159	468.01
GEO	−7.7049***	1	−4.8058**	280.60***
KAZ	−3.7557**	1	−9.7245***	287.82***
KYR	−2.5929	1	−7.9850***	118.26***
LAT	−1.7788	1	−7.6466***	144.96***
LIT	−1.8872	1	−9.2086***	36.601***
MOL	−3.2999*	2	−3.6051	64.014
RF	−0.4181	1	−4.5355**	135.54***
TJK	−2.2166	1	−9.9251***	55.479***
TKM	−2.9547	2	−1.7443	142.52
UA	−3.8328**	1	−1.9917	91.419
UZB	−9.5218***	1	−7.0085***	605.52***

*Note*: ADF = augmented Dickey–Fuller test; FADF = Fourier augmented Dickey–Fuller test (Becker *et al.*, 2006). C&T = model specification with constant and trend. *k* indicates the lag length selected using the Akaike information criterion (AIC) and Bayesian information criterion (BIC). *F* denotes the Fourier frequency test statistic. ***, **, and * indicate rejection of the null hypothesis of a unit root at the 1%, 5%, and 10% significance levels, respectively.

According to the ADF test results, the series for Armenia (ARM), Azerbaijan (AZE), Estonia (EST), Georgia (GEO), Kazakhstan (KAZ), Moldova (MOL), Ukraine (UA), and Uzbekistan (UZB) were found to be stationary. This implies that in these countries, IMRs tend towards a certain level over time, and shocks do not have permanent effects in the long run. This implies that in these countries, IMRs remain stationary around a certain equilibrium level over time, and shocks do not have permanent effects in the long run.

Alternatively, the Fourier ADF test,^1^ which accounts for structural breaks, provides a more flexible stationarity analysis. Based on the Fourier ADF test results, the series for Armenia, Azerbaijan, Georgia, Kazakhstan, Kyrgyzstan (KYR), Latvia (LAT), Lithuania (LIT), Russian Federation (RF), Tajikistan (TJK), and Uzbekistan are stationary. These findings indicate that the Matthew Effect hypothesis holds true in these countries; that is, IMRs tend toward a certain level over time, reinforcing inequality.

For robustness checks, various unit root tests in addition to the ADF and FADF tests were conducted. The detailed results of these tests are presented in Appendix, Tables A4–A8. According to the results of the KSS, Sollis, and Kruse tests, the null hypothesis was rejected for Armenia, Azerbaijan, Georgia, Kazakhstan, Kyrgyzstan, Latvia, Lithuania, Moldova, Tajikistan, Turkmenistan, Ukraine, and Uzbekistan, concluding that the series in these countries are stationary (see Appendix, Table A4–A6). Moreover, the Fractional Flexible Fourier ADF test showed that the null hypothesis of stationarity was rejected for all the countries mentioned above except Armenia (see Appendix, Table A8). Due to the statistical insignificance of Fourier terms, the test results for Armenia are invalid, and thus a definitive assessment for this country could not be made.

**Figure 2.**
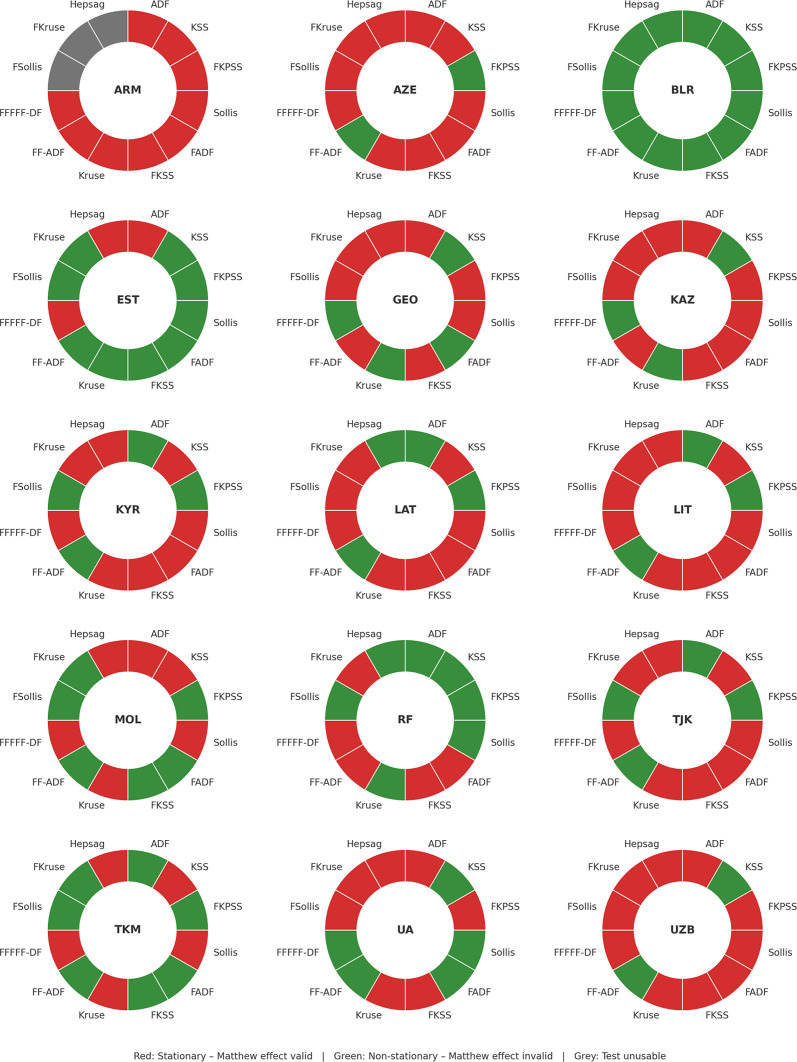
Multitest evaluation of infant mortality rates across 15 post-Soviet countries.

Based on the Fourier KSS test (Christopoulos and León-Ledesma, 2010), stationarity was confirmed for Armenia, Azerbaijan, Georgia, Kazakhstan, Kyrgyzstan, Latvia, Lithuania, Russian Federation, Tajikistan, and Uzbekistan (see Appendix, Table A4). Meanwhile, the Fourier Kruse test indicated that IMR series are stationary for all the mentioned countries except Armenia (see Appendix, Table A6). The results obtained from the Fourier Kruse test for Armenia were considered invalid due to statistically insignificant Fourier terms. Although the IMR series appears stationary according to the FSollis test, the associated F-statistic is not statistically significant. In FSollis, the F-statistic evaluates the joint significance of the sine and cosine Fourier terms that model smooth structural changes in the data. If these terms are found to be statistically insignificant or effectively zero, it suggests that the series does not exhibit the type of structural variation that the Fourier specification is intended to capture. Since FSollis is an extended version of the classical Sollis test that incorporates Fourier components, the insignificance of these terms implies that the model essentially reduces to the standard Sollis form. In such cases, it becomes methodologically more appropriate to interpret the results using the classical Sollis test rather than its Fourier-based version. This interpretive rule also applies to other Fourier-based unit root tests: when the test indicates stationarity, the statistical significance of the F-statistic must be checked to ensure that the Fourier terms meaningfully contribute to the model. However, if the test fails to reject the null hypothesis of non-stationarity, the F-statistic becomes irrelevant, and no further validation of the Fourier terms is required. Based on the same test, Azerbaijan, Lithuania, and Latvia are identified as stationary, whereas Belarus, Estonia, Georgia, Kazakhstan, Kyrgyzstan, Moldova, the Russian Federation, Tajikistan, Turkmenistan, Ukraine, and Uzbekistan are found to be non-stationary. In addition, the Fourier KPSS (FKPSS) test results indicated that the null hypothesis of stationarity was rejected for all countries. Since the FKPSS test operates under a reversed hypothesis structure – where the null assumes stationarity and the alternative assumes non-stationarity – this result implies that the infant mortality series in all countries are non-stationary. Consequently, this finding suggests that the Matthew Effect hypothesis is not valid for these countries, as persistent inequality cannot be empirically confirmed. It is essential to interpret FKPSS results with special care due to this reversed null formulation. Furthermore, for Armenia, the Fourier terms were found to be statistically insignificant, meaning that the Fourier approximation does not contribute to the test’s explanatory power. Therefore, the FKPSS results for Armenia are considered invalid, and no definitive conclusion regarding the stationarity of its IMR series can be drawn based on this test. On the other hand, the Flexible Fourier ADF test identified stationarity only for Ukraine and Uzbekistan (see Appendix, Table A7). Lastly, according to the Hepsag (2021) test results, the null hypothesis was rejected for Armenia, Azerbaijan, Estonia, Georgia, Kazakhstan, Kyrgyzstan, Lithuania, Tajikistan, Turkmenistan, Ukraine, and Uzbekistan (see Appendix, Table A8). However, the null hypothesis could not be rejected for Belarus, Latvia, and Russian Federation, indicating that the Matthew Effect hypothesis does not hold for these countries. All the unit root test results discussed above, including both traditional and Fourier-based approaches, are synthesized and visually summarized in Figure 2 for a comprehensive comparative evaluation. The donut charts display the proportion of stationary and non-stationary outcomes from the 12 unit root tests. In these visualizations, green segments represent non-stationary outcomes (indicating a lack of support for the Matthew Effect), while red segments represent stationary outcomes (suggesting its presence). The radar plots, meanwhile, illustrate the year-to-year evolution of IMRs.

The reason why the Fourier Sollis (Ranjbar *et al.,* 2018), Fourier Kruse (Güriş, 2019), and Hepsag (2021) tests produced invalid results for Armenia can be primarily attributed to the nature of the structural break observed in the series. Unlike the smooth and gradual shifts that these Fourier-based tests are designed to capture, the IMR series for Armenia exhibits a sharp and abrupt structural break, which significantly undermines the statistical power and reliability of these tests.

**Figure A1.**
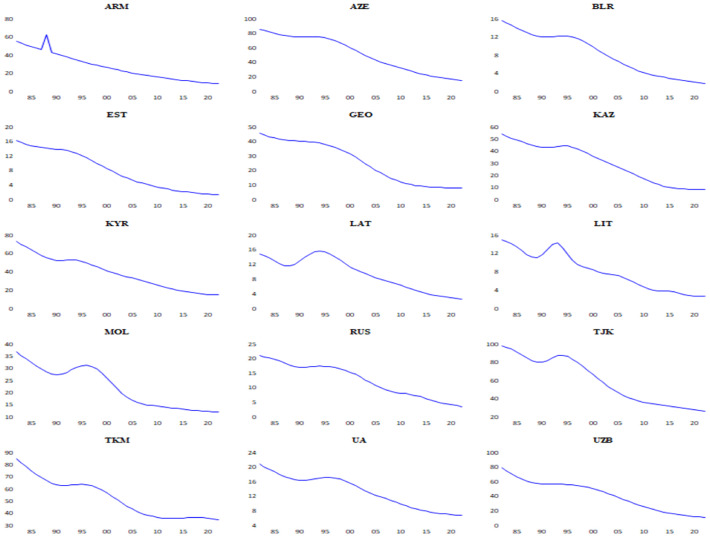
Time series graphics of IMR by countries.

This abrupt change is historically linked to the 1988 Spitak earthquake, a catastrophic event that caused massive human loss, including a substantial number of infant deaths. As illustrated in the Appendix, Table A1 and Figure A1, the trend in Armenia sharply deviates in 1988: while the IMR in 1987 was recorded at 47 deaths per 1,000 live births, it abruptly increased to 63 in 1988, only to return to its previous trajectory in 1989. This single-year spike represents a distinct and sudden structural shock, rather than a smooth transition, rendering the Fourier approximation ineffective for capturing the nature of the break.

In contrast, other post-Soviet countries in the sample display smoother transitions or trends in infant mortality, making them more suitable for analysis using Fourier-based frameworks. Consequently, for Armenia, traditional unit root tests such as the standard Sollis test provide more valid insights.

In order to assess the strength and structural intensity of the Matthew Effect across countries, three different multi-criteria decision-making methods were applied: Equal Weighting, Entropy Weighting, and TOPSIS. Each of these methods aggregates the binary outcomes of 12 unit root tests – either confirming or rejecting stationarity – based on its own weighting logic. Using the composite scores generated by MCDA, the 15 post-Soviet countries were classified into three categories: Weak, Moderate, and Strong levels of the Matthew Effect. This classification is presented in the Table 4, which not only shows the relative strength of the Matthew Effect across countries but also reveals the level of consistency and divergence among the different weighting approaches. Notably, the countries in the Moderate category exhibit greater variation between methods, underscoring the importance of using multiple evaluation techniques for a more reliable policy interpretation.

**Table 4.** MCDA-based Matthew effect strength classification Table 4 long description.

Country	Equal weighting	Classification	Entropy weighting	Classification	TOPSIS	Classification		
ARM	0.76	Strong	0.79	Strong	0.70	Strong	Mattew Effect Score	Strong (≥0.68)
AZE	0.83	Strong	0.65	Moderate	0.47	Moderate		
BLR	0.00	Weak	0.00	Weak	0.00	Weak		
EST	0.25	Weak	0.18	Weak	0.26	Weak		
GEO	0.66	Moderate	0.74	Strong	0.69	Strong		
KAZ	0.75	Strong	0.81	Strong	0.73	Strong		Moderate (0.34–0.67)
KYR	0.67	Moderate	0.46	Moderate	0.37	Moderate		
LAT	0.67	Moderate	0.52	Moderate	0.42	Moderate		
LIT	0.75	Strong	0.56	Moderate	0.43	Moderate		
MOL	0.50	Moderate	0.37	Moderate	0.36	Moderate		
RF	0.42	Moderate	0.41	Moderate	0.47	Moderate		Weak (<0.34)
TJK	0.67	Moderate	0.46	Moderate	0.37	Moderate		
TKM	0.42	Moderate	0.28	Weak	0.30	Weak		
UA	0.58	Moderate	0.56	Moderate	0.51	Moderate		
UZB	0.83	Strong	0.72	Strong	0.55	Moderate		

Legend: Table 4 reports the strength of the Matthew Effect in infant mortality across 15 post-Soviet countries using three MCDA approaches: Equal Weighting, Entropy Weighting, and TOPSIS. Scores are classified into three categories: **Strong (≥0.68)**, **Moderate (0.34–0.67)**, and **Weak (<0.34)**, as indicated in the colour legend on the right-hand side. Higher scores imply stronger persistence of infant mortality inequality consistent with the Matthew Effect.

*Source:* Authors’ calculations.

**Figure 3.**
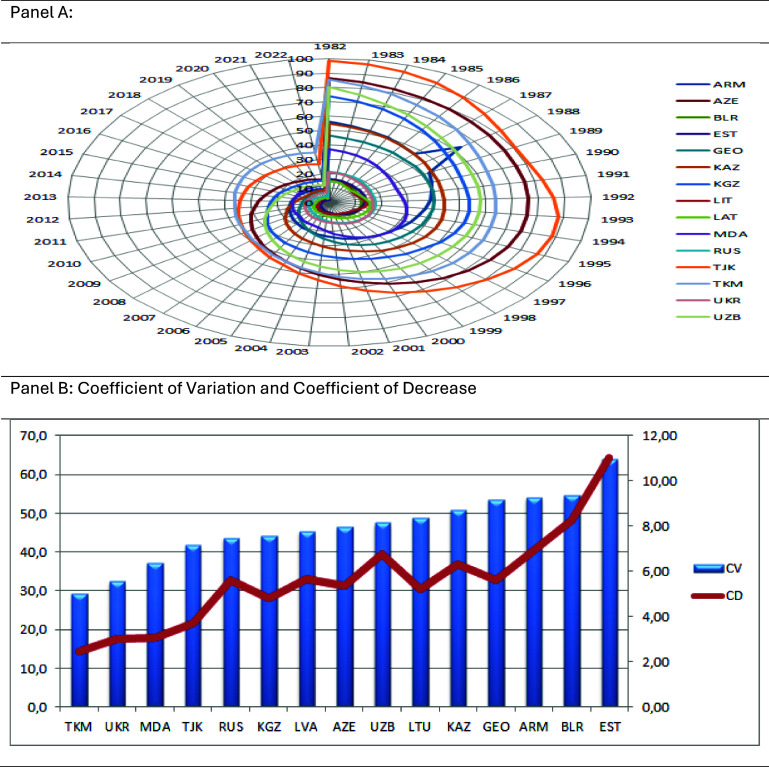
Figure 3 long description. Comparison of infant mortality trends using the radar chart, the coefficient of variation (CV) and the coefficient of decrease (CD).

To further enrich the interpretation of the findings, Figure 3 combines two complementary visualizations: a bar chart of the coefficient of variation (CV) and coefficient of decrease (CD), and country-specific radar charts displaying the temporal patterns of IMRs. The CV measures the extent of variability in infant mortality over time; higher values suggest large fluctuations, while lower values imply more stable trends. The CD, on the other hand, quantifies the overall speed of decline; a high CD indicates a rapid reduction in mortality rates, whereas a low CD points to stagnation or minimal progress. Countries with low CV and high CD exhibit patterns of convergence and saturation, aligning with the Matthew Effect. In contrast, high CV and low CD values reveal volatile or inconsistent trajectories, suggesting that the series has not stabilized. The radar charts visually reinforce these conclusions: inwardly contracting patterns signal sustained declines, while outward or irregular shapes reflect instability. Together, these descriptive visuals complement the econometric results and enhance the overall analytical narrative.

Within the scope of the tests conducted above, the country-based evaluation in terms of the Matthew Effect is presented below:

For Armenia, the time series on the spiral graph starts at a medium level and steadily declines over time. The high CV and the significantly meaningful CD value indicate a notable improvement in infant mortality in the country. While the Matthew Effect scores calculated with three different weighting methods (0.76, 0.79, and 0.70) point to a strong effect, the fact that all series were found to be stationary in unit root tests shows that this effect is also methodologically valid. Therefore, Armenia is one of the countries where the Matthew Effect is strongly observed.

Although Azerbaijan achieved improvement after starting with high IMRs, the downward trend observed in the spiral graph is not as sharp as in other countries. While the CV and CD values remain at a medium-high level, the equally weighted Matthew Effect score is striking at 0.83. The scores calculated by the other two methods are at more moderate levels (0.65 and 0.47). The fact that all series were found to be stationary in unit root analyses shows that the Matthew Effect is also methodologically valid. In this context, a strong but relatively limited Matthew Effect is observed in Azerbaijan.

In the evaluation made for Belarus, despite the regular decline observed in the spiral graph, it is striking that all Matthew Effect scores obtained under three criteria are zero (0.00). This situation suggests that the improvement observed in the country is not related to an initial advantage but can rather be explained by structural or external factors. Indeed, the fact that the series were found to be non-stationary in unit root tests reveals that the Matthew Effect is statistically invalid in this country. Therefore, despite showing positive development, Belarus should be considered invalid in terms of the Matthew Effect.

Estonia (EST), having already had low IMRs at the beginning, was in an advantageous position and consolidated this advantage over time with its curve in the spiral graph not deviating from the centre. However, despite the CV and CD values being at the highest levels, the very low Matthew Effect scores obtained under three different weighting methods (0.25, 0.18, and 0.26) show that this advantage cannot be technically confirmed within the context of the Matthew Effect. The fact that the series were found to be non-stationary in most of the unit root tests also supports this interpretation. Therefore, the positive performance observed in Estonia is based not on the Matthew Effect, but on the initial advantage.

For Georgia, the spiral graph starts at a medium level and the improvement curve becomes more apparent over time. Especially the high entropy and TOPSIS weighted scores (0.74 and 0.69) indicate that a relative Matthew Effect has occurred in the country. Additionally, the CV and CD values also indicate a parallel development. The fact that the series were found to be stationary in most unit root tests supports this effect. In this context, Georgia demonstrates a moderate but methodologically valid Matthew Effect.

Kazakhstan draws a relatively rapid improvement curve from a high starting point on the spiral graph. Strong Matthew Effect scores were calculated in all three different metrics (0.75, 0.81, 0.73), and in most unit root tests, the series were found to be stationary. Considering the high CV and CD values, the progress observed in Kazakhstan represents a development model where the classical Matthew Effect is valid.

For Kyrgyzstan, the spiral graph points to higher starting points, with a limited decline observed over time. The fact that all scores calculated according to three different weighting methods remain at a “moderate” level shows that the Matthew Effect is weak but observable in the country. Although mixed results were obtained in unit root tests, stationarity is predominant. Therefore, it can be said that a moderate Matthew Effect is valid in Kyrgyzstan.

Lithuania is one of the countries that has attracted attention with low IMRs from the beginning. The spiral graph shows that this advantage has been preserved over time. Although the equally weighted Matthew Effect score is high at 0.75, the scores remain at a moderate level in the other two metrics, revealing that the initial advantage has been preserved but positive accumulation is limited. In addition, most of the series were found to be non-stationary in unit root tests. In this context, the positive development observed in Lithuania is not technically supported within the scope of the Matthew Effect.

Latviasimilarly has a low initial level and shows a regular decline in the spiral graph. However, the fact that all Matthew Effect scores remain at a moderate level (0.67, 0.52, 0.42) and that mixed results were found in unit root tests shows that the effect occurred at a limited level in this country. Latvia demonstrates a moderate Matthew Effect along with positive development.

For Moldova, the spiral graph shows a more average starting point and a gradual decline. The scores also remain at a moderate level (0.50, 0.37, 0.36), indicating that the improvement in infant mortality is not based on a distinct Matthew Effect. In the unit root tests, since stationarity is predominant, the effect can be considered methodologically valid. Moldova is among the countries where a limited but valid Matthew Effect is observed.

Specifically for Russia, the spiral graph points to a high starting point and a slow decline. The fact that the scores remain at a moderate level and that unit root tests yield mixed but stationarity-leaning results indicate a weak but technically supported Matthew Effect in the country.

Tajikistan is one of the countries with the highest starting level in the graphs and exhibits the slowest decline on the spiral graph. Nevertheless, moderate level Matthew Effect scores were obtained in all three metrics. Moreover, the fact that the series were found to be stationary in all unit root tests shows that this effect is technically valid. Therefore, Tajikistan, as one of the most disadvantaged countries, is one of the rare examples where the Matthew Effect is methodologically valid despite limited improvement.

Turkmenistan similarly has a high initial value and shows rather slow development over time. Especially the low entropy weighted score (0.28) and mixed results in unit root tests indicate a weak Matthew Effect in the country.

For Ukraine, the spiral graph shows a medium-level starting point and a steady decline. Moderate scores were obtained in all three metrics. Although some of the tests in the unit root analysis found the series to be non-stationary, the general tendency is toward stationarity. Therefore, a moderate but valid Matthew Effect can be mentioned for Ukraine.

Uzbekistan exhibits a high starting point and a relatively rapid decline in the spiral graph. High Matthew Effect scores were obtained in all three weightings (0.83, 0.72, 0.55). The fact that the series were found to be stationary in unit root tests methodologically supports this strong effect. Uzbekistan, despite having a high starting point, is one of the countries showing the most significant improvement in infant mortality and is one of the strongest examples where the Matthew Effect is valid.

Therefore, the findings reveal that in several countries infant mortality has reached a certain level and become stationary, while in others it continues to fluctuate over time. In our study, the series were found to be stationary for Armenia, Azerbaijan, Georgia, Kazakhstan, Kyrgyzstan, Lithuania, Latvia, Tajikistan, Turkmenistan, Ukraine, and Uzbekistan, confirming the presence of the Matthew Effect in the sample of these countries. It is understood that infant mortality in these countries has reached a certain level over time and has not shown major changes. It is concluded that the improvement in infant mortality has stagnated after a certain point in these countries. Compared to previous studies, the non-stationary findings obtained for Belarus, Estonia, and the Russian Federation are consistent with Yılancı and Canpolat Gökçe (2021). On the other hand, the results obtained for Moldova, Latvia, and Ukraine in this study differ from the findings reached by Yılancı and Canpolat Gökçe (2021). These different results may arise from the analysed period, the methods used, or changes in health policies over time.

Additionally, it was found that the series were not stationary for Belarus, Estonia, Moldova, and Russia. This result indicates that the IMR has not yet reached a stable long-term level and that dynamic fluctuations persist. This shows that the Matthew Effect does not hold in these countries, meaning that the accumulated advantages in health indicators did not persist with the same intensity. The difficulty of health policies in surpassing this level shows that the health system in these countries is stuck at a certain threshold and that further progress cannot be achieved without additional interventions. These findings are in line with previous studies, such as Bishai *et al.* (2007); and Aguirre and Vela-Peón (2015) found that the Matthew Effect does not hold.

## Conclusion and policy recommendations

This study investigates the stationarity of IMRs in 15 post-Soviet countries using a comprehensive range of unit root tests, including traditional, non-linear, and Fourier-based approaches. The primary objective was to evaluate the validity of the Matthew Effect, which posits that initial advantages in health outcomes tend to persist and accumulate over time. Our findings demonstrate that the IMR series are stationary in the majority of countries, implying that health inequalities are not only enduring but potentially self-reinforcing. In contrast, a smaller set of countries exhibited non-stationary behaviour, suggesting ongoing transitions or volatility in their infant mortality dynamics.

The presence of stationarity in countries such as Armenia, Azerbaijan, Georgia, Kazakhstan, Kyrgyzstan, Lithuania, Latvia, Tajikistan, Turkmenistan, Ukraine, and Uzbekistan suggests that IMRs in these nations has remain stationary at a certain threshold and has not shown major changes. In line with the Matthew Effect, these countries appear to have reached a saturation point in policy effectiveness – one that requires more complex, targeted, and high-investment interventions to achieve further gains. Conversely, non-stationary countries such as Belarus, Estonia, Moldova, and the Russian Federation reflect patterns of policy inconsistency, external shocks, or socio-political disruptions that prevent sustainable reductions in IMRs.

From a policy perspective, the implications are clear: to break the cycle of persistent inequality in infant mortality, governments must not rely solely on broad-based or static health interventions. Rather, they should adopt a multidimensional, equity-focused policy framework that targets the root causes of infant mortality and recognizes its socio-economic determinants. One of the most critical interventions lies in women’s education. Extensive empirical evidence demonstrates that maternal education significantly reduces the likelihood of infant death by improving health knowledge, increasing access to care and enhancing decision-making within households. Thus, expanding access to quality education for girls – particularly in rural and marginalized areas – should be a national priority.

Closely related is the issue of early marriage, which remains prevalent in several post-Soviet states, such as Azerbaijan, Kyrgyzstan, Georgia, and Tajikistan. These countries have a higher rate of early marriage compared to other post-Soviet countries. In 2023, in regard to the population of women aged 20–24, the percentage of those who married before the age of 18 was almost 15% in Azerbaijan, 9.4% in Georgia, and 9% in Kyrgyzstan. In Tajikistan, this proportion was 9% in 2017^2^ (The Child Marriage Data Portal, 2025). Early childbearing not only increases the biological risks associated with pregnancy and childbirth but also curtails educational and economic opportunities for young women. Policymakers in the aforementioned nations must implement legal frameworks to postpone the marriage age and provide alternative pathways through education, vocational training, and youth empowerment programmes.

Moreover, access to clean drinking water, sanitary living conditions, and hygiene infrastructure must be ensured, especially in underserved and remote regions. Waterborne diseases and poor sanitation are among the leading indirect causes of infant mortality. Thus, investments in community-based water filtration systems, regular health inspections, and hygiene awareness campaigns are indispensable, particularly in rural areas of Azerbaija, Tajikistan, Turkmenistan, Kyrgyzstan, and Uzbekistan, where access to safe water and sanitation remains limited. Nutrition is another pillar. Pregnant women must be supported not only to eat to satisfy hunger but to achieve adequate nutritional intake that meets the demands of fetal development. This need is especially significant in Tajikistan, Kyrgyzstan, Armenia, Moldova, and Uzbekistan, where nutritional deficiencies among pregnant women are more widespread. Governments should establish maternal nutrition programmes that include access to micronutrient supplements, fortified foods, prenatal vitamins, and where necessary, subsidized financial support to purchase essential items. Community-based nutritional counselling and mobile health services could also serve as vital outreach mechanisms. Additionally, universal antenatal care and postnatal services must be strengthened and decentralized. Ensuring that every pregnancy is monitored by qualified health professionals reduces complications during delivery and improves neonatal outcomes. This can be achieved through targeted subsidies for maternal care, incentives for healthcare professionals to work in rural areas, and the integration of digital health technologies to support remote monitoring. These measures are particularly pertinent for Armenia, Georgia, Azerbaijan, Kazakhstan, and Moldova, where inequalities in rural maternal healthcare endure, whereas the advancement of digital health is most viable in Estonia, Lithuania, Latvia, and Azerbaijan, due to their more advanced e-health infrastructures.

In countries, like Belarus, Moldova, Russia, and Armenia, where health reforms have stagnated or fragmented, institutional reforms should prioritize system-wide coherence, ensuring that maternal and child health services are not siloed but embedded within a larger continuum of care. Multi-sectoral coordination among the ministries of health, education, infrastructure, and social welfare is essential throughout the region, particularly in countries like Tajikistan, Kyrgyzstan, Moldova, and Armenia, where weak social protection systems intensify health disparities. Finally, for populations experiencing extreme poverty or displacement, direct cash transfers, food assistance programmes, and conditional support schemes tied to health service utilization may provide the safety net needed to reduce infant deaths. These should be complemented by robust monitoring and evaluation systems to assess effectiveness and adjust interventions dynamically. This approach is especially pertinent for Ukraine, where war has resulted in significant relocation, as well as for Georgia, Moldova, Tajikistan, and Kyrgyzstan, where escalated poverty levels and vulnerable people exacerbate infant death rates.

Consequently, the sustained decline in the IMR in the post-Soviet region cannot be explained solely by economic growth or technological innovation. As the literature shows, infant mortality can be influenced by broader social determinants such as women’s education and empowerment, access to primary and maternal and child health services, social protection, and equality. Therefore, the stationary or weak dynamics of the IMR in some post-Soviet countries may indicate that the development process is not sufficiently inclusive. In this sense, for faster progress across the region, it is important to design social policies in a more inclusive manner, in line with country-specific institutional and social realities, in addition to strengthening health infrastructure.

The findings of this study reveal that sustainable health policies are critical for addressing infant mortality, and that countries’ progress in the field of health should be monitored in the long term. In particular, in stationary countries, the effectiveness of existing health policies should be enhanced, while in non-stationary countries, more stable and long-term health reforms should be implemented to reduce fluctuations. Future studies will be able to achieve more precise results by examining the impact of changes in health policies on infant mortality using larger datasets.

Addressing the limitations of this study is crucial, since they may guide further research:

- This research employs several unit root tests to figure out the current state. The impact of variables such as health policy measures and socio-economic determinants on infant mortality was not explicitly examined in this study. Stationarity tests provide insights into persistence, although they do not identify the particular reasons of mortality reductions or the efficacy of measures.
- The study uses aggregate national data, ignoring regional and socio-economic variation at the country level. IMRs tend to demonstrate significant gaps between the rural and the urban environment, as well as between different socio-economic and ethnic groups. Country-level time series also form an irreplaceable component when conducting cross-country comparisons, but they cannot provide for the full intra-country variability.
- This research does not model the factors that influence infant mortality. Hence, the effects of global and regional events impacting infant mortality, such the COVID-19 pandemic, economic crisises, the Russia–Ukraine and the Karabakh wars, were not considered individually.

In conclusion, future research should address these constraints by utilizing regional and household-level data, employing alternative econometric techniques, and explicitly incorporating the influence of health expenditure, education, and institutional quality.

## Data Availability

The datasets used and/or analysed during the current study are publicly available from World Development Indicators (World Bank, 2025, https://databank.worldbank.org/source/world-development-indicators).

## Footnotes

1
The critical values for the constant and trended equation of the FADF test are given in Appendix, Table A3.

2
For Tajikistan, this data is available until 2017.

## Appendix

**Table A1.** Trend analysis Table A1 long description.

Panel A: Trend analysis results		
Countries	C	Trend
ARM	4.1174***	−0.0469***
AZE	4.7212***	−0.0450***
BLR	3.0219***	−0.0549***
EST	3.1474***	−0.0659***
GEO	4.1253***	−0.0520***
KAZ	4.2780***	−0.0514***
KYR	4.3738***	−0.0403***
LAT	2.9939***	−0.0428***
LIT	2.8850***	−0.0450***
MOL	3.6527***	−0.0309***
RUS	3.2752***	−0.0420***
TJK	4.7363***	−0.0367***
TKM	4.3992***	−0.0236***
UKR	3.1203***	−0.0288***
UZB	4.5436***	−0.0470***

*** implies the signifigance of constant and trend.

**Table A2.** Linearity test results Table A2 long description.

Countries	Harvey and Leybourne (2007)			Harvey *et al.* (2008)
	W*_10%	W*_5%	W*_1%	W_lam
ARM	16.63*	16.74**	16.94***	2.39
AZE	23.82*	24.06**	24.49***	4.47
BLR	65.82*	66.49**	67.70***	7.12**
EST	37.26*	37.63**	38.29***	3.33
GEO	19.49*	19.68**	20.01***	5.31*
KAZ	12.88*	13.00**	13.22	4.93*
KYR	25.07*	25.26**	25.61***	4.97*
LAT	25.11*	25.38**	25.87***	2.79
LIT	18.97*	19.12**	19.40***	3.51
MOL	11.00*	11.09**	11.25	5.58*
RF	28.21*	28.49**	28.99***	2.11
TJK	14.67*	14.80**	15.04***	4.10
TKM	8.78*	8.84	8.96	6.04**
UA	13.88*	14.00**	14.22***	5.14*
UZB	52.96*	53.46**	54.37***	4.37

***, **, and * indicate that the null hypothesis of linearity is rejected at 1%, 5%, and 10% significance levels, respectively. For a chi-square distribution with **4 degrees of freedom**, the critical values at the **10%**, **5%**, and **1%** significance levels are **7.78**, **9.49**, and **13.28**, respectively. In the case of **2 degrees of freedom**, the corresponding critical values are **4.61**, **5.99**, and **9.21**.

**Table A3.** Critical values for the constant and trended equation of the FADF test Table A3 long description.

	*k*	1%	5%	10%
*T* = 100	1	−5.11	−4.46	−4.15
	2	−4.83	−4.16	−3.79
	3	−4.50	−3.83	−3.49
	4	−4.39	−3.70	−3.36
	5	−4.37	−3.63	−3.28
*T* = 250	1	−4.93	−4.34	−4.06
	2	−4.72	−4.04	−3.72
	3	−4.44	−3.80	−3.45
	4	−4.26	−3.67	−3.33
	5	−4.22	−3.59	−3.25
*T* = 500	1	−4.86	−4.30	−4.02
	2	−4.64	−4.02	−3.69
	3	−4.39	−3.77	−3.46
	4	−4.24	−3.64	−3.32
	5	−4.13	−3.59	−3.27

*Note:* The critical values presented in this table are sourced from Hepsağ (2022), who generated them specifically for the Fourier ADF test with both constant and trend specifications. This choice is justified by the fact that all IMR series analysed in this study exhibit a statistically significant trend component, as confirmed by the trend analysis results presented in Appendix 1, Table A1 and Figure A1.

**Table A4.** Results of KSS and Fourier KSS Table A4 long description.

Countries	KSS	FKSS		
	C&T	C&T		
	Test stat	*k*	Test stat	*F*
ARM	−4.0788***	1	−4.2608***	23.035***
AZE	−4.8256***	1	−6.0556***	645.25***
BLR	−2.1953	1	−1.2330	305.83
EST	−1.7478	1	−2.6151	468.01
GEO	−5.4262***	1	−4.1042**	280.60***
KAZ	−4.3899***	1	−7.3053***	287.82***
KYR	−3.9964***	1	−5.7111***	118.26***
LAT	−3.4736**	1	−7.1571***	144.96***
LIT	−5.2601***	1	−6.2893***	36.601***
MOL	−5.9609***	2	−3.6285	64.014
RF	−1.4621	1	−4.7424**	135.54***
TJK	−4.1422***	1	−9.1338***	55.479***
TKM	−6.2034***	2	2.7212	142.52***
UA	−3.3899*	1	−2.0836	91.419***
UZB	−6.7656***	1	−6.2818***	605.52***

***, **, and * indicate that the null hypothesis is rejected at 1%, 5%, and 10% significance level, respectively.

**Table A5.** Results of Sollis and Fourier Sollis Table A5 long description.

Countries	Sollis	FSollis		
	C&T	C&T		
	Test stat	*k*	Test stat	*F*
ARM	8.3569**	1	10.486**	2.1231
AZE	17.085***	1	45.305**	355.41***
BLR	2.3468	1	0.4640	252.28
EST	1.5281	1	2.1142	896.21
GEO	14.645***	1	0.4811	2072.6
KAZ	11.587***	1	0.6930	481.14
KYR	9.0593***	1	0.7659	52.016
LAT	5.8916*	1	30.038***	54.352***
LIT	13.610***	1	32.511***	19.239***
MOL	25.352***	2	5.6325	261.13***
RF	2.1055	1	0.8729	83.599
TJK	8.8570***	2	4.6465	74.485
TKM	25.114***	1	7.2421	667.13
UA	6.9351**	1	0.6010	76.431
UZB	27.782***	1	0.3241	843.53

***, **, and * indicate that the null hypothesis is rejected at 1%, 5%, and 10% significance level, respectively.

**Table A6.** Results of Kruse and Fourier Kruse Table A6 long description.

Countries	Kruse	FKruse		
	C&T	C&T		
	Test stat	*k*	Test stat	*F*
ARM	17.703***	1	20.038**	3.0351
AZE	27.364***	1	35.741***	645.27***
BLR	4.7263	1	1.6145	305.83
EST	3.6802	1	6.8745	468.01
GEO	30.753***	1	16.471*	280.60***
KAZ	21.989***	1	52.636***	287.82***
KYR	17.650***	1	34.833***	118.26***
LAT	11.742*	1	52.582***	144.96***
LIT	28.294***	1	44.360***	36.601***
MOL	45.914***	2	13.048	64.014
RF	3.7988	1	22.120**	135.54***
TJK	16.928**	1	82.107***	55.479***
TKM	45.371***	2	14.476	142.52
UA	15.192**	1	5.2694	91.419
UZB	54.481***	1	39.462***	605.52***

***, **, and * indicate that the null hypothesis is rejected at 1%, 5%, and 10% significance level, respectively.

**Table A7.** Results of Fourier KPSS and flexible Fourier ADF Table A7 long description.

Countries	FKPSS			FFADF		
	C&T			C&T		
	*k*	Test stat	*F*	*k*	Test stat	*F*
ARM	1	0.0984***	3.0351	5	0.4253	0.7306
AZE	1	0.0755***	645.25	1	0.2437	5.8955**
BLR	1	0.0770***	305.83	1	0.1244	17.810***
EST	1	0.0723***	468.01	1	0.1269	22.209***
GEO	1	0.0706**	280.60	1	0.0497	10.865***
KAZ	1	0.0996***	287.82	1	−2.1060	6.4126**
KYR	1	0.1211***	118.26	1	−2.6909	6.1319**
LAT	1	0.0848***	144.96	3	−0.0703	11.835***
LIT	1	0.1657***	36.601	4	−0.0952	6.5364**
MOL	2	0.1206***	64.014	2	−2.7467	12.925***
RF	1	0.0809***	135.54	2	0.6442	2.9249
TJK	1	0.0780***	55.479	2	−2.1087	12.144***
TKM	2	0.1201***	142.52	2	−2.8176	69.084***
UA	1	0.0806***	91.419	1	−6.1715***	46.384***
UZB	1	0.0744***	605.52	1	−9.0976***	291.73***

***, and ** indicate that the null hypothesis is rejected at 1%, and 5% significance level, respectively.

**Table A8.** Results of fractional flexible Fourier ADF and Hepsağ Table A8 long description.

Countries	FFFFF ADF			Hepsağ (2021)		
	C&T			C&T		
	*k*	Test stat	*F*	Test stat	BP	BT
ARM	0.2	−4.7102***	1.7376	20.253***	**1.5460**	
AZE	0.2	−8.3732***	23.366***	17.519***	0.3166	1994
BLR	0.2	−2.4907	12.299	3.9796	0.4952	2001
EST	0.2	−3.6097*	17.453***	35.480***	0.4193	1998
GEO	0.2	−4.8715****	2.2494	19.120***	0.6234	2007
KAZ	0.2	−6.1794***	9.6983***	16.757**	0.7945	2014
KYR	0.2	−5.3937***	10.716***	12.649**	0.2668	1992
LAT	0.2	−10.728***	43.675***	4.8378	0.2374	1991
LIT	0.2	−8.6552***	22.774***	45.380***	0.5779	2005
MOL	0.2	−10.843***	16.328***	25.791***	0.5175	2002
RF	0.2	−4.8654***	10.060***	1.4774	0.3342	1995
TJK	0.2	−9.7330***	23.439***	64.429***	0.5040	2002
TKM	0.2	−13.264***	10.378***	24.716***	0.5823	2005
UA	0.2	−3.8319*	5.4493**	11.595***	0.6053	2006
UZB	0.2	−8.4167***	10.639***	11.302***	0.3235	2004

***, **, and * indicate that the null hypothesis is rejected at 1%, 5%, and 10% significance level, respectively. FFFFF, Fractional Frequency Flexible Fourier Form.

## Figure 1. Long description

Panel A: A line graph showing the infant mortality rate per 1,000 live births in Armenia, Azerbaijan, Belarus, Estonia, Georgia, Kazakhstan, Kyrgyz Republic, Latvia, Lithuania, Moldova, Russian Federation, Tajikistan, Turkmenistan, and Uzbekistan from 1990 to 2023. The horizontal axis represents the years from 1990 to 2023, and the vertical axis represents the mortality rate ranging from 0 to 90 per 1,000 live births. Each country is represented by a distinct colored line. The graph shows a general downward trend in infant mortality rates across all countries over the years. Specific trends for each country include Armenia showing a steady decline, Azerbaijan with a significant drop, and Turkmenistan with a notable decrease. Other countries like Kazakhstan and Kyrgyz Republic also exhibit declining trends, while some countries like Estonia and Latvia maintain relatively lower mortality rates throughout the period.

Navigate back to Figure 1..

## Table 1. Long description

A table with three columns and one row. The columns are labeled Variable, Definition, Period, and Source. The row provides details for the IMR variable. Under Variable, it states IMR. Under Definition, it states Infant mortality rate (per 1,000 live births). Under Period, it states 1982-2022. Under Source, it states World Bank, World Development Indicators (World Bank, 2025).

Navigate back to Table 1..

## Table 2. Long description

The table presents descriptive statistics for IMRs across various countries. It includes columns for Mean, Median, Minimum, Maximum, Standard Deviation, and Jarque-Bera probability values. The table has 15 rows, each representing a different country, and 7 columns for the statistical measures. Row 1: ARM, 27.9, 24.6, 9.19, 63.4, 15.1, 3.43(0.18). Row 2: AZE, 52.2, 53.6, 16.1, 86.5, 24.3, 4.31(0.12). Row 3: BLR, 8.32, 8.50, 1.90, 15.7, 4.54, 4.01(0.13). Row 4: EST, 8.16, 7.30, 1.50, 16.5, 5.23, 4.37(0.11). Row 5: GEO, 26.2, 27.5, 8.30, 46.4, 14.0, 4.81(0.09). Row 6: KAZ, 30.6, 32.5, 8.69, 54.8, 15.6, 3.97(0.14). Row 7: KYR, 39.5, 38.4, 15.4, 74.0, 17.5, 2.48(0.20). Row 8: LAT, 9.66, 10.3, 2.80, 15.9, 4.39, 3.64(0.16). Row 9: LIT, 8.35, 7.90, 2.90, 15.1, 4.08, 3.39(0.18). Row 10: MDA, 22.3, 21.8, 12.2, 37.1, 8.26, 4.37(0.11). Row 11: RF, 12.8, 13.9, 3.80, 21.3, 5.59, 3.74(0.15). Row 12: TJK, 60.1, 58.4, 26.7, 98.6, 25.1, 4.63(0.10). Row 13: TKM, 52.9, 51.6, 35.0, 85.5, 15.5, 3.21(0.20). Row 14: UKR, 13.5, 14.3, 7.00, 21.1, 4.39, 3.40(0.18). Row 15: UZB, 42.5, 47.1, 11.9, 80.4, 20.2, 2.76(0.25).

Navigate back to Table 2..

## Table 3. Long description

A table comparing the results of ADF and Fourier ADF tests across various countries. The table has 14 rows and 5 columns. The columns are labeled as Countries, ADF Test stat, FADF (2010) k, FADF (2010) Test stat, and FADF (2010) F. The row labels are country codes. Each row provides the test statistics for the ADF and FADF tests, along with the value of k for the FADF test. Row 1: ARM, -4.3112***, 1, -4.6943**, 23.035***. Row 2: AZE, -5.3783***, 1, -7.0944***, 645.25***. Row 3: BLR, -2.9723, 1, -1.3679, 305.83. Row 4: EST, -3.9890**, 1, -1.7159, 468.01. Row 5: GEO, -7.7049***, 1, -4.8058**, 280.60***. Row 6: KAZ, -3.7557**, 1, -9.7245***, 287.82***. Row 7: KYR, -2.5929, 1, -7.9850***, 118.26***. Row 8: LAT, -1.7788, 1, -7.6466***, 144.96***. Row 9: LIT, -1.8872, 1, -9.2086***, 36.601***. Row 10: MOL, -3.2999*, 2, -3.6051, 64.014. Row 11: RF, -0.4181, 1, -4.5355**, 135.54***. Row 12: TJK, -2.2166, 1, -9.9251***, 55.479***. Row 13: TKM, -2.9547, 2, -1.7443, 142.52. Row 14: UA, -3.8328**, 1, -1.9917, 91.419. Row 15: UZB, -9.5218***, 1, -7.0085***, 605.52***.

Navigate back to Table 3..

## Table 4. Long description

The table presents the classification of 15 post-Soviet countries based on the strength of the Matthew Effect using three methods: Equal Weighting, Entropy Weighting, and TOPSIS. The table has 15 rows and 7 columns. The columns are labeled as Country, Equal Weighting, Classification, Entropy Weighting, Classification, TOPSIS, and Classification. The rows are labeled with country codes. The values in the columns represent composite scores and classifications into three categories: Weak, Moderate, and Strong. The table shows the relative strength of the Matthew Effect across countries and the level of consistency and divergence among the different weighting approaches. Notably, the countries in the Moderate category exhibit greater variation between methods.

Navigate back to Table 4..

## Figure 3. Long description

Panel A: A radar chart displays the dynamics of infant mortality rates (IMRs) across various countries from 1982 to 2022. The chart includes data for countries such as ARM, AZE, BLR, EST, GEO, KAZ, KGZ, LTU, LVA, MDA, RUS, TKM, TJK, UKR, and UZB. Each country is represented by a different colored line, showing the trend of IMRs over the years. Panel B: A combination of a bar graph and a line graph illustrates the coefficient of variation (CV) and the coefficient of decrease (CD) for different countries. The bar graph, with blue bars, represents the CV values, while the line graph, with a red line, represents the CD values. The horizontal axis lists countries such as TKM, UKR, MDA, TJK, RUS, KGZ, LVA, AZE, UZB, LTU, KAZ, GEO, ARM, BLR, and EST. The vertical axis on the left measures the CV values ranging from 0 to 70, and the vertical axis on the right measures the CD values ranging from 0 to 12.

Navigate back to Figure 3..

## Table A1. Long description

A table titled ‘Panel A: Trend analysis results’ with two columns labeled ‘Countries’ and ‘Trend’. The table contains data for 18 countries. The first column lists the country codes, and the second column shows the trend values. Row 1: ARM, 4.1174***, -0.0469***. Row 2: AZE, 4.7212***, -0.0450***. Row 3: BLR, 3.0219***, -0.0549***. Row 4: EST, 3.1474***, -0.0659***. Row 5: GEO, 4.1253***, -0.0520***. Row 6: KAZ, 4.2780***, -0.0514***. Row 7: KYR, 4.3738***, -0.0403***. Row 8: LAT, 2.9939***, -0.0428***. Row 9: LIT, 2.8850***, -0.0450***. Row 10: MOL, 3.6527***, -0.0309***. Row 11: RUS, 3.2752***, -0.0420***. Row 12: TJK, 4.7363***, -0.0367***. Row 13: TKM, 4.3992***, -0.0236***. Row 14: UKR, 3.1203***, -0.0288***. Row 15: UZB, 4.5436***, -0.0470***.

Navigate back to Table A1..

## Table A2. Long description

A table comparing linearity test results across countries using different methods. The table has 15 rows and 5 columns. The columns are labeled ‘Countries’, ‘Harvey and Leybourne (2007) W*_10%’, ‘Harvey and Leybourne (2007) W*_5%’, ‘Harvey and Leybourne (2007) W*_1%’, and ‘Harvey et al. (2008) W_lam’. The row labels are country codes. Each cell contains a numerical value representing the test results. Notable trends include varying values across different countries and methods, with some countries showing higher values in specific columns.

Navigate back to Table A2..

## Table A3. Long description

A table comparing critical values for constant and trended equations of the FADF test. The table has three main sections based on different values of T (100, 250, 500) and each section contains rows for k values ranging from 1 to 5. Each row lists critical values at the 1 percent, 5 percent, and 10 percent levels. Row 1: k 1, 1 percent -5.11, 5 percent -4.46, 10 percent -4.15. Row 2: k 2, 1 percent -4.83, 5 percent -4.16, 10 percent -3.79. Row 3: k 3, 1 percent -4.50, 5 percent -3.83, 10 percent -3.49. Row 4: k 4, 1 percent -4.39, 5 percent -3.70, 10 percent -3.36. Row 5: k 5, 1 percent -4.37, 5 percent -3.63, 10 percent -3.28. Row 6: k 1, 1 percent -4.93, 5 percent -4.34, 10 percent -4.06. Row 7: k 2, 1 percent -4.72, 5 percent -4.04, 10 percent -3.72. Row 8: k 3, 1 percent -4.44, 5 percent -3.80, 10 percent -3.45. Row 9: k 4, 1 percent -4.26, 5 percent -3.67, 10 percent -3.33. Row 10: k 5, 1 percent -4.22, 5 percent -3.59, 10 percent -3.25. Row 11: k 1, 1 percent -4.86, 5 percent -4.30, 10 percent -4.02. Row 12: k 2, 1 percent -4.64, 5 percent -4.02, 10 percent -3.69. Row 13: k 3, 1 percent -4.39, 5 percent -3.77, 10 percent -3.46. Row 14: k 4, 1 percent -4.24, 5 percent -3.64, 10 percent -3.32. Row 15: k 5, 1 percent -4.13, 5 percent -3.59, 10 percent -3.27.

Navigate back to Table A3..

## Table A4. Long description

The table presents the results of KSS and FKSS tests for various countries. It has 15 rows and 5 columns. The columns are labeled as Countries, KSS Test stat, FKSS k, FKSS Test stat, and FKSS F. The row labels are the country codes. The values in the columns represent the test statistics and the number of Fourier terms used in the FKSS test. Row 1: ARM, -4.0788***, 1, -4.2608***, 23.035***. Row 2: AZE, -4.8256***, 1, -6.0556***, 645.25***. Row 3: BLR, -2.1953, 1, -1.2330, 305.83. Row 4: EST, -1.7478, 1, -2.6151, 468.01. Row 5: GEO, -5.4262***, 1, -4.1042**, 280.60***. Row 6: KAZ, -4.3899***, 1, -7.3053***, 287.82***. Row 7: KYR, -3.9964***, 1, -5.7111***, 118.26***. Row 8: LAT, -3.4736**, 1, -7.1571***, 144.96***. Row 9: LIT, -5.2601***, 1, -6.2893***, 36.601***. Row 10: MOL, -5.9609***, 2, -3.6285, 64.014. Row 11: RF, -1.4621, 1, -4.7424**, 135.54***. Row 12: TJK, -4.1422***, 1, -9.1338***, 55.479***. Row 13: TKM, -6.2034***, 2, 2.7212, 142.52***. Row 14: UA, -3.3899*, 1, -2.0836, 91.419***. Row 15: UZB, -6.7656***, 1, -6.2818***, 605.52***.

Navigate back to Table A4..

## Table A5. Long description

A table comparing test statistics and F-values for different countries using Sollis and Fourier Sollis methods. The table has 14 rows and 6 columns. The columns are labeled as Countries, Sollis C&T Test stat, FSollis k, FSollis C&T Test stat, and FSollis C&T F. The row labels are country codes. Each row provides the test statistic for Sollis C&T, the number of lags k for FSollis, and the test statistic and F-value for FSollis C&T. Notable values include high test statistics for AZE, GEO, MOL, TKM, and UZB in the Sollis C&T column, and high F-values for AZE, GEO, MOL, TKM, and UZB in the FSollis C&T column.

Navigate back to Table A5..

## Table A6. Long description

A table comparing test statistics for various countries using Kruse and Fourier Kruse methods. The table has 14 rows and 5 columns. The columns are labeled as Countries, Kruse Test stat, FKruse k, FKruse Test stat, and FKruse F. The row labels are the country codes. The values in the table are as follows: Row 1: ARM, 17.703***, 1, 20.038**, 3.0351. Row 2: AZE, 27.364***, 1, 35.741***, 645.27***. Row 3: BLR, 4.7263, 1, 1.6145, 305.83. Row 4: EST, 3.6802, 1, 6.8745, 468.01. Row 5: GEO, 30.753***, 1, 16.471*, 280.60***. Row 6: KAZ, 21.989***, 1, 52.636***, 287.82***. Row 7: KYR, 17.650***, 1, 34.833***, 118.26***. Row 8: LAT, 11.742*, 1, 52.582***, 144.96***. Row 9: LIT, 28.294***, 1, 44.360***, 36.601***. Row 10: MOL, 45.914***, 2, 13.048, 64.014. Row 11: RF, 3.7988, 1, 22.120**, 135.54***. Row 12: TJK, 16.928**, 1, 82.107***, 55.479***. Row 13: TKM, 45.371***, 2, 14.476, 142.52. Row 14: UA, 15.192**, 1, 5.2694, 91.419. Row 15: UZB, 54.481***, 1, 39.462***, 605.52***.

Navigate back to Table A6..

## Table A7. Long description

A table comparing Fourier KPSS and flexible Fourier ADF test statistics across countries. The table has 15 rows and 6 columns. The columns are labeled as Countries, FKPSS C&T k, FKPSS C&T Test stat, FKPSS C&T F, FFADF C&T k, and FFADF C&T Test stat. The row labels are country names. Each row provides values for k, Test stat, and F under FKPSS C&T and k and Test stat under FFADF C&T. The table includes data for countries such as ARM, AZE, BLR, EST, GEO, KAZ, KYR, LAT, LIT, MOL, RF, TJK, TKM, UA, and UZB. The values vary across countries and tests, indicating different levels of stationarity and non-stationarity.

Navigate back to Table A7..

## Table A8. Long description

A table comparing unit root test results for various countries using the FFFFF ADF and Hepsağ methods. The table has 15 rows and 8 columns. The columns are labeled as follows: Countries, k, Test stat, F, Test stat, BP, and BT. The row labels are country codes: ARM, AZE, BLR, EST, GEO, KAZ, KYR, LAT, LIT, MOL, RF, TJK, TKM, UA, and UZB. Each row provides values for the respective country under the FFFFF ADF C&T and Hepsağ C&T methods. The table includes statistical test results and break points for each country.

Navigate back to Table A8..
